# *Candida lusitaniae* as an Unusual Cause of Recurrent Vaginitis and its Successful Treatment With Intravaginal Boric Acid

**DOI:** 10.1155/S1064744901000400

**Published:** 2001

**Authors:** Neil S. Silverman, Margie Morgan, W. S. Nichols

**Affiliations:** 1 Department of Obstetrics and Gynecology Cedars-Sinai Medical Center Burns & Allen Research Institute UCLA School of Medicine Los Angeles CA 90048 USA; 2 Department of Pathology and Laboratory Medicine Cedars-Sinai Medical Center Burns & Allen Research Institute UCLA School of Medicine Los Angeles CA USA

## Abstract

Increasing use of short-course antifungal therapies in patients with recurrent vulvovaginitis may enable the emergence of less-common, more resistant yeast strains as vaginal pathogens. We report the case of a patient with chronically symptomatic and repeatedly treated vaginal candidiasis whose infection was attributable to *Candida* *lusitaniae*, a previously unreported cause of candidal vaginitis .


*Infect Dis Obstet Gynecol 2001;9:245–247*

### *Candida lusitaniae* as an unusual cause of recurrent vaginitis and its successful treatment with intravaginal boric acid

**Neil S. Silverman[1], Margie Morgan[2] and W.S. Nichols[2]**

[1]*Department of Obstetrics and Gynecology, Cedars-Sinai Medical Center, Burns & Allen Research Institute, UCLA School of Medicine, Los Angeles, CA*
[2]*Department of Pathology and Laboratory Medicine, Cedars-Sinai Medical Center, Burns & Allen Research Institute, UCLA School of Medicine, Los Angeles, CA*

Increasing use of short-course antifungal therapies in patients with recurrent vulvovaginitis may enable the emergence of less-common, more resistant yeast strains as vaginal pathogens. We report the case of a patient with chronically symptomatic and repeatedly treated vaginal candidiasis whose infection was attributable to *Candida lusitaniae*, a previously unreported cause of candidal vaginitis.

Key words: VULVOVAGINITIS; VAGINITIS; CANDIDA; BORIC ACID

### CASE REPORT

A 55-year-old gravida 4, para 2022 Caucasian woman was referred for evaluation and management of chronic recurrent vulvovaginitis. The patient reported approximately five years of vaginal symptoms consisting primarily of 'burning' and 'itching', with 'outbreaks' occurring every 1–3 months. She had been treated, in response to symptoms and culture results, with a variety of systemic and topical antimicrobials and antifungals over the past years. Her most recent antifungal therapy was 6 months prior to referral, and consisted of a two-dose course of oral fluconazole with moderate symptom relief. Approximately 1 month prior to referral, the patient had been treated with a seven-day course of intravaginal clindamycin cream after a vaginal culture was positive for *Gardnerella vaginalis*; she reported only 1–2 days of symptomatic relief after that therapy.

The patient was in good health, with no chronic medical conditions. She was taking no medications. A total abdominal hysterectomy with ovarian preservation had been performed 17 years earlier for uterine leiomyomata. The patient reported no menopausal vasomotor symptoms, and was not taking hormone replacement therapy. Evaluation of follicle-stimulating hormone (FSH) and luteinizing hormone (LH) levels had recently been performed in her referring doctor's office and were not in the menopausal range.

At the time of initial evaluation, the patient reported only minimal symptomatology. Examination revealed no redness or swelling of the external genitalia or the vagina. No lesions or excoriations were present. The vaginal pH was 4.5, and microscopic examination of the vaginal secretions showed only normal-appearing epithelial cells with scattered large rod-form bacteria. No

Correspondence to: Neil S. Silverman, Department of Obstetrics and Gynecology, Cedars-Sinai Medical Center, 8700 Beverly Boulevard, Suite 160W, Los Angeles, CA 90048. Email: silvermann@cshs.org treatment was instituted empirically, and vaginal cultures obtained at that visit subsequently returned negative both for yeast and pathogenic bacteria. The patient was instructed to return on symptom recurrence.

The patient returned for symptom recurrence two months later. Examination was unremarkable, vaginal pH was 4.5, and wet prep again showed normal-appearing epithelial cells, without evidence of yeast or other pathogens. Vaginal cultures were obtained with the fungal culture positive for yeast, with speciation pending. Bacterial cultures were negative. The patient was started on oral fluconazole to begin at 200 mg every 4 days for three doses as 'induction' therapy, with a plan to begin chronic suppressive therapy if eradication of symptoms and colonization were proven on follow-up[1]. The patient returned after completing her initial treatment, reporting that her symptoms had improved for two days after the first dose, but had then returned and were still present. Repeat examination showed the presence of budding yeast on saline prep, and a course of oral ketoconazole, 100 mg/day, was started. Five days after starting the ketoconazole, the speciation results from the first positive yeast culture returned as *Candida lusitaniae*, with the more recent cultures again positive for yeast (these subsequently were also identified as *C. lusitaniae*). The yeast were identified in our laboratory through morphology on cornmeal agar (Hardy Diagnostics, Santa Maria, CA) and inoculation of an API 20 C assimilation strip (bioMerieux, Hazelwood, MO). To eliminate confusion with other yeast species, a maltose fermentation test was performed in the laboratory too, with absence of maltose fermentation confirming the presence of *C. lusitaniae*. The patient was contacted and reported that she was still symptomatic, with no relief from the ketoconazole. Her treatment was changed to boric acid vaginal suppositories, 600 mg nightly for 14 days. The patient's symptoms resolved, and a follow-up vaginal fungal culture two weeks after completing therapy was negative. She developed a reddened, irritated site at the posterior forchette toward the end of therapy, which responded to a topical emollient cream.

### DISCUSSION

While *Candida albicans* remains the most common yeast species implicated in symptomatic vulvovaginitis, recent reports have described a relative decrease in its proportional impact compared with a relative rise in disease attributable to non-*albicans* species. One recent review calculated an increase in non-*albicans* vaginal infections from 10% in the 1970s to 21% through the 1980s, with *Torulopsis glabrata* and *C. tropicalis* demonstrating the largest percentage increases for individual species[2,3].

The change in breakdown of pathogenic yeast species has significant implications for the treatment of candidal vulvovaginitis. The widening diversity in identified yeast strains is thought to result at least in part from expanding use of short-course topical imidazole antifungals, allowing for the emergence of resistant organisms. *C. tropicalis* and *T. glabrata*, for example, have been shown to be resistant to a variety of topical preparations both *in vitro* and *in vivo*[4,5], and *C. tropicalis* has cell-wall characteristics that make it intrinsically less susceptible to imidazole compounds[5]. Shorter courses of less-effective regimens may also allow for overgrowth of more resistant intravaginal yeast species, as has been shown to be the case with the use of low-dose antifungal prophylaxis against systemic yeast infections in neutropenic or immunosuppressed patients[6]. While the shorter-course therapies for vulvovaginitis may enhance compliance for some patients, they may be insufficient or even detrimental for those women with recurrent symptoms who are then treated with multiple sequential courses of both topical and systemic antifungals, allowing the emergence of less-sensitive non-*albicans* species.

The present case describes the identification of *C. lusitaniae* as the pathogen responsible for the most recent episodes of recurrent vulvovaginitis in a chronically-treated woman, and underscores the increased need to consider such uncommon fungal strains in refractory cases. *C. lusitaniae* was first identified as an opportunistic human pathogen in 1979 in a patient with acute leukemia[7], and has been identified as a source of fungemia in only 42 cases reported through 2000[8,9]. Two-thirds of these cases occurred in immunocompromised patients, and no cases of vaginitis attributable to *C. lusitaniae* in otherwise healthy women have been reported to date. Of note is the fact that *C. lusitaniae* has been associated with breakthrough fungemia in patients being treated with single-agent antifungal regimens, though recent investigators have suggested that, in their experience, the use of fluconazole might be an effective single-agent approach against this pathogen in immunocompetent fungemic patients[8].

Both fluconazole and ketoconazole proved clinically and microbiologically ineffective against the *C. lusitaniae* vaginitis seen in our patient. Both agents have been shown to be effective in a high proportion of women with recurrent vulvovaginal candidiasis, though non-*albicans* species, primarily *T. glabrata*, have been reported to be more difficult to control even with longer-duration regimens of these systemic agents[1,10]. The use of boric acid to treat vulvovaginal candidiasis was first reported in 1974[11], and it has been shown to be effective against refractory non-*albicans* yeast species, specifically *T. glabrata*[12]. Presented in this case with a non-*albicans* species associated with a similarly high degree of imidazole resistance, a treatment regimen of intravaginal boric acid capsules as described in Sobel's series was employed, with both symptomatic and bacteriologic cure against *C. lusitaniae* achieved.

In reporting what we believe to be the first reported case of recurrent vaginitis attributable to *C. lusitaniae*, along with its successful treatment with non-azole therapy, we add our concerns to others' over the rise of less-common, more difficult-to-treat yeast species in patients with chronically treated infections. While our patient ultimately responded to intravaginal boric acid therapy, this case demonstrates the value of yeast speciation in difficult recurrent cases, and the ongoing need to investigate newer antifungal therapies in the face of an increasingly diverse spectrum of pathogenic agents, even in immunocompetent individuals.

### REFERENCES

1. Sobel JD. Vulvovaginits. When *Candida* becomes a problem. *Derm Clin NA* 1998;16:763–8
2. Odds FC, Webster CE, Riley VC, *et al.* Epidemiology of vaginal *Candida* infection: significance of numbers of vaginal yeasts and their biotypes. *Eur J Obstet Gynecol Reprod Biol* 1987; 25:53–66
3. Horowitz BJ, Giaquinta D, Ito S. Evolving pathogens in vulvovaginal candidiasis: implications for patient care. *J Clin Pharmacol* 1992;32:248–55
4. Kerridge D, Nicholas RO. Drug resistance in the opportunistic pathogens *Candida albicans* and *Candida glabrata. J Antimicrob Chemother* 1986;18 (suppl B):39–49
5. Horowitz BJ. Candidiasis: speciation and therapy. *Curr Prob Obstet Gynecol Fertil* 1990;8:241–5
6. Meunier-Carpentier F, Kiehn JR, Armstrong D. Fungemia in the immunocompromised host: changing patterns, antigenemia, high mortality. *Am J Med* 1981;71:363–70
7. Pappagianis D, Collins MS, Hector R, *et al.* Development of resistance to amphotericin B in *Candida lusitaniae* infecting a human. *Antimicrob Agents Chemother* 1979;16:123–6
8. Minari A, Hachen R, Raad I. *Candida lusitaniae*: a cause of breakthrough fungemia in cancer patients. *Clin Infect Dis* 2001;32:186–90
9. Blinkhorn RJ, Adelstein D, Spagnuolo PJ. Emergence of a new opportunistic pathogen, *Candida lusitaniae. J Clin Microbiol* 1989;27:236–40
10. Sobel JD. Recurrent vulvovaginal candidiasis: a prospective study of the efficacy of maintenance ketoconazole therapy. *N Engl J Med* 1986;315: 1455–8
11. Swate TE, Weed JC. Boric acid treatment of vulvovaginal candidiasis. *Obstet Gynecol* 1974;43: 893–5
12. Sobel JD, Chaim W. Treatment of *Torulopsis glabrata* vaginitis: retrospective review of boric acid therapy. *Clin Infect Dis* 1997;24:649–52

RECEIVED 06/18/01; ACCEPTED 10/04/01
